# Pulsed‐Field Ablation for Atrial Fibrillation in Partial Anomalous Pulmonary Venous Return With Right Atrial Drainage of Both Right Pulmonary Veins

**DOI:** 10.1002/joa3.70485

**Published:** 2026-09-28

**Authors:** Keisho Kurokawa, Hiroshi Kawakami, Tomoki Fujisawa, Momo Taira, Rikako Horie, Shunsuke Tamaki, Osamu Yamaguchi

**Affiliations:** ^1^ Department of Cardiology, Pulmonology, Hypertension and Nephrology Ehime University Graduate School of Medicine Toon Japan; ^2^ Department of Medical Support Ehime University Hospital Toon Japan

**Keywords:** adult congenital heart disease, atrial fibrillation, partial anomalous pulmonary venous return, pulsed‐field ablation

## Abstract

Pulsed‐field ablation was safely performed in a patient with atrial fibrillation and partial anomalous pulmonary venous return. Electroanatomical mapping defined the sinus node region and confirmed sufficient distance from anomalous right pulmonary vein ablation sites, enabling successful isolation.

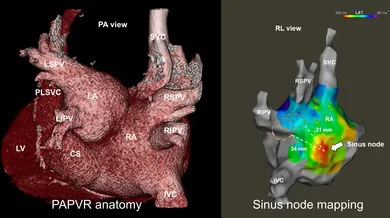

## 1 Introduction

Partial anomalous pulmonary venous return (PAPVR) is a rare congenital anomaly [1], and catheter ablation for atrial fibrillation (AF) in this setting remains challenging because of complex anatomy. Although several case series have reported catheter ablation in PAPVR, data on pulsed‐field ablation (PFA) remain limited.

PFA enables myocardial‐selective ablation through irreversible electroporation and has emerged as a promising modality for pulmonary vein isolation [2]. However, in cases where anomalous pulmonary veins drain into the right atrium, ablation may be performed in close proximity to the sinus node, raising potential safety concerns, particularly regarding sinus node injury.

Herein, we report a case of persistent AF with PAPVR in which both right pulmonary veins drained into the right atrium, successfully treated with PFA guided by electroanatomical mapping with functional localization of the sinus node.

## 2 Case Presentation

A 78‐year‐old man with known partial anomalous pulmonary venous return (PAPVR) and persistent left superior vena cava developed symptomatic persistent atrial fibrillation (AF) with heart failure. Echocardiography and right heart catheterization showed marked right ventricular dilatation with a significant left‐to‐right shunt (Qp/Qs 2.36). Surgical repair of PAPVR was planned; however, catheter ablation was performed first to achieve rhythm control for heart failure associated with symptomatic persistent AF. Cardiac computed tomography demonstrated that both the right superior and inferior pulmonary veins drained directly into the right atrium without an atrial septal defect (Figure 1).

**FIGURE 1:**
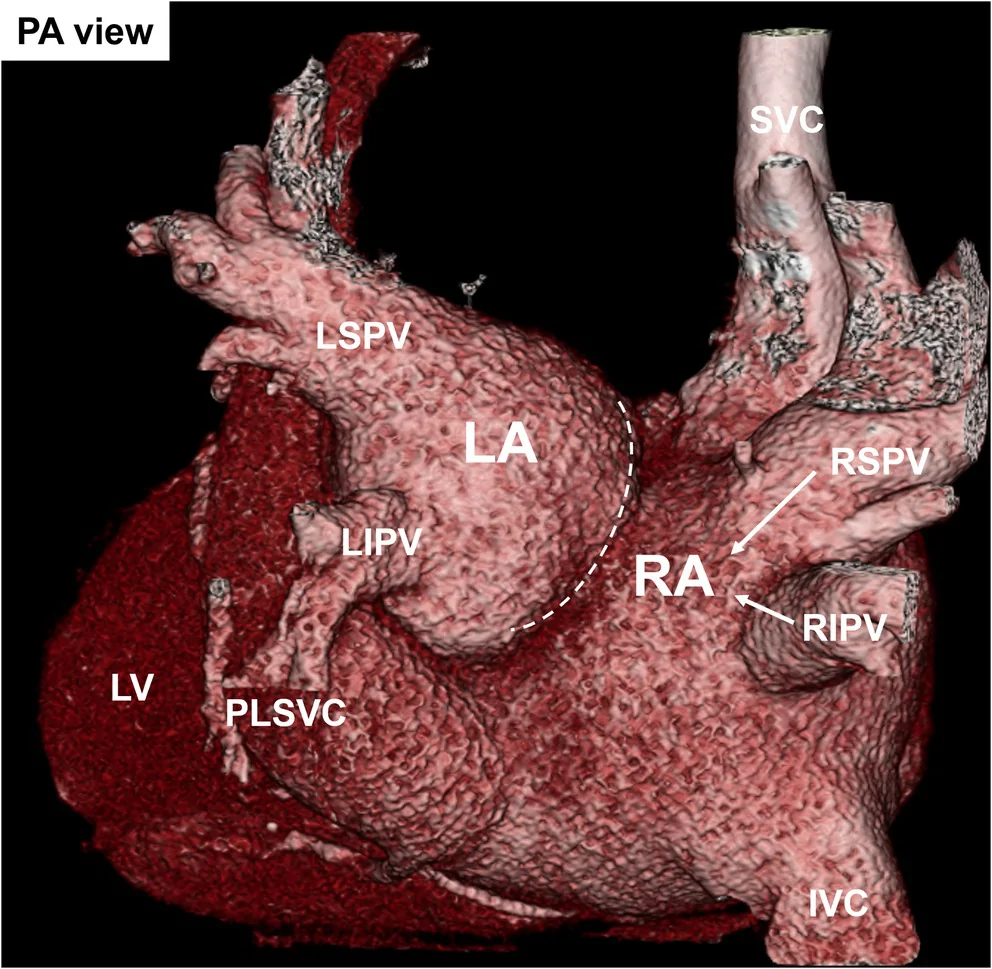
Computed tomography‐based three‐dimensional reconstruction showing anomalous drainage of the right superior and right inferior pulmonary veins into the right atrium. The left pulmonary veins drain normally into the left atrium. IVC, inferior vena cava; LA, left atrium; LIPV, left inferior pulmonary vein; LSPV, left superior pulmonary vein; LV, left ventricle; PLSVC, persistent left superior vena cava; RA, right atrium; RIPV, right inferior pulmonary vein; RSPV, right superior pulmonary vein; SVC, superior vena cava.

Catheter ablation using pulsed‐field ablation (PFA) was performed. After electrical cardioversion restored sinus rhythm, electroanatomical mapping of the right atrium and anomalous pulmonary veins was performed using a high‐density mapping catheter (Octaray, Biosense Webster, Irvine, CA, USA). Three‐dimensional mapping delineated the anatomical relationships, and preserved electrical potentials were identified at the antral regions of both anomalous pulmonary veins (Figure 2A). The SVC and ostial‐to‐mid PLSVC were mapped and showed local electrical potentials, but no spontaneous ectopic or trigger activity originated from either structure; therefore, neither was isolated. Pulmonary vein isolation was first performed for the left pulmonary veins using a pulsed‐field ablation system (PulseSelect, Medtronic, Minneapolis, MN, USA). Electrical potentials were recorded at the ostial and antral regions of both anomalous right pulmonary veins, although no spontaneous ectopic activity was documented. Given the preserved electrical connections and their potential arrhythmogenic role in PAPVR, both anomalous right pulmonary veins were targeted for isolation. Before PFA delivery to the anomalous right pulmonary veins, three‐dimensional mapping enabled functional localization of the sinus node and demonstrated that the planned ablation sites were 31 and 34 mm away from the sinus node region (Figure 3A). High‐output pacing at 10 V was also performed to assess right phrenic nerve capture, which was observed only at distal sites. The PulseSelect catheter was then positioned in the right atrium, and PFA was delivered from the right atrial side to isolate both anomalous right pulmonary veins. Pre‐ and post‐ablation bipolar voltage maps in the same right lateral view are shown in Figure 3B,C, respectively. PFA applications were delivered to the LSPV, LIPV, anomalous RSPV, and anomalous RIPV in 15, 12, 11, and 8 applications, respectively, based on four distal and four proximal applications per vein, with additional antral or carinal applications as needed. No sinus node dysfunction or phrenic nerve injury occurred. Final remapping confirmed complete isolation of all pulmonary veins (Figure 2B). A 24‐h Holter recording at 1 month showed sinus rhythm without AF recurrence or pauses ≥ 3 s, with maximum, mean, and minimum heart rates of 93, 70, and 45 bpm, respectively, suggesting no clinically apparent sinus node dysfunction. Thereafter, serial electrocardiograms obtained at approximately monthly outpatient visits showed sinus rhythm until surgical repair of PAPVR 6 months after catheter ablation.

**FIGURE 2:**
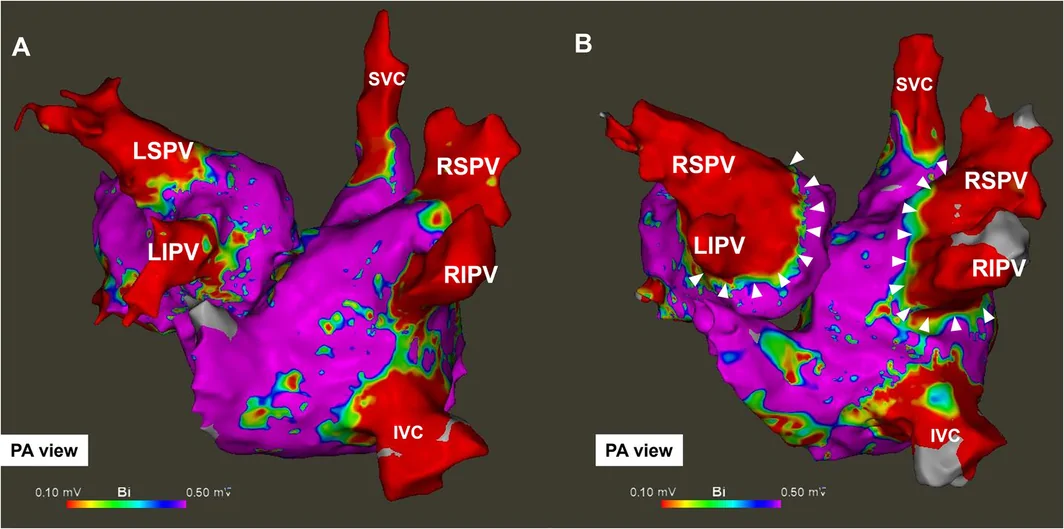
Three‐dimensional electroanatomical voltage maps of the right and left atria, superior vena cava, and pulmonary veins in the posteroanterior view. (A) Pre‐ablation showing preserved antral potentials. (B) Post‐ablation demonstrating electrical isolation of both left and anomalous right pulmonary veins. IVC, inferior vena cava; LA, left atrium; LIPV, left inferior pulmonary vein; LSPV, left superior pulmonary vein; RA, right atrium; RIPV, right inferior pulmonary vein; RSPV, right superior pulmonary vein; SVC, superior vena cava.

**FIGURE 3:**
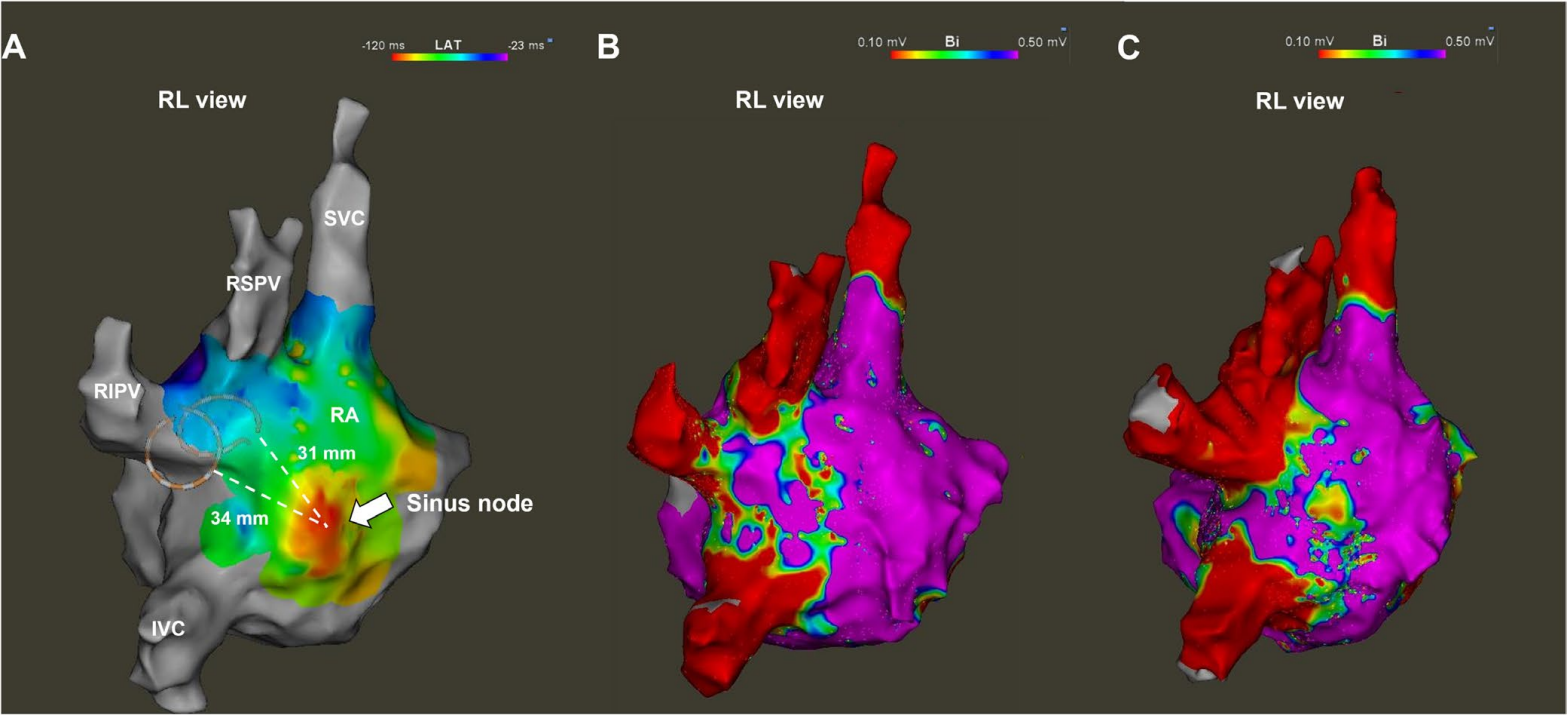
Right lateral view of three‐dimensional electroanatomical maps. (A) Local activation time map during sinus rhythm showing the sinus node region (white arrow) and the PulseSelect catheter positions at the antral regions of the right superior and right inferior pulmonary veins during pulsed‐field ablation. The distances from the sinus node region to the right superior and right inferior pulmonary vein ablation sites were 31 and 34 mm, respectively. (B, C) Pre‐ and post‐ablation bipolar voltage maps obtained in the same projection and displayed with the same voltage scale. The post‐ablation map demonstrates isolation of the anomalous right pulmonary veins, while the low‐voltage area adjacent to the sinus node region was already present before ablation. IVC, inferior vena cava; RA, right atrium; RIPV, right inferior pulmonary vein; RL, right lateral; RSPV, right superior pulmonary vein; SVC, superior vena cava.

## 3 Discussion

PAPVR with direct drainage of both right pulmonary veins into the right atrium is an extremely rare anatomical variant and poses unique challenges for AF ablation. Approximately 59% of anomalous pulmonary veins drain into the superior vena cava, whereas direct drainage into the right atrium accounts for only about 12% of cases [1]. Although several reports have described catheter ablation in PAPVR, data on pulsed‐field ablation (PFA) remain limited, and prior PFA cases have involved drainage into the superior vena cava [3].

In this anatomical setting, myocardial tissue selectivity of PFA does not necessarily ensure sinus node protection. Therefore, electroanatomical mapping was used to define the spatial relationship between the sinus node region and the intended PFA delivery sites [4]. The sinus node region was 31 and 34 mm from the anomalous right superior and inferior pulmonary vein ablation sites, respectively, and no clinically apparent sinus node dysfunction was observed during or after the procedure. However, in the present case, sinus node activation was not reassessed at higher heart rates using pharmacological stimulation or atrial pacing. Because the earliest sinus activation site may shift with changes in heart rate and autonomic tone [5], such assessment could provide a more comprehensive evaluation of the safety margin, particularly when PFA delivery is planned near the sinus node region. In addition, the PulseSelect system, with its over‐the‐wire design, may facilitate stable tissue contact and contribute to procedural safety in complex anatomy [6].

The pathophysiological role of anomalous pulmonary veins in AF remains incompletely understood. Electrical activity within anomalous pulmonary veins has been reported in approximately 35% of PAPVR cases, and isolation of such veins has been associated with a reduced risk of AF recurrence [7]. These findings support the importance of evaluating and isolating electrically active anomalous pulmonary veins in selected patients.

Although the present patient had persistent AF, and atrial remodeling may have contributed to arrhythmogenesis, sinus rhythm was maintained during short‐term follow‐up [8]. However, this is a single case with limited follow‐up, and no conclusions can be drawn regarding long‐term efficacy.

This case demonstrates that PFA can be safely performed in PAPVR with complex anatomy when guided by electroanatomical mapping with functional localization of the sinus node.

## Funding

The authors have nothing to report.

## Ethics Statement

Ethical approval was waived by the Ethics Committee of Ehime University Graduate School of Medicine.

## Consent

Written informed consent was obtained from the patient for publication of this case report.

## Conflicts of Interest

The authors declare no conflicts of interest.

## Data Availability

The data that support the findings of this study are available from the corresponding author upon reasonable request.
